# Understanding the Nexus Between Anxiety and Acoustic Perception in University Students: A Quasi-Experimental Study During Pandemic-Induced Lockdown

**DOI:** 10.3390/bs15030262

**Published:** 2025-02-24

**Authors:** Lingjiang Huang, Jialin Li, Jian Kang, Fangfang Liu, Ming Yang, Yawei Zhang

**Affiliations:** 1 School of Urban Design, Wuhan University, Wuhan 430072, China; huanglj@whu.edu.cn (L.H.); 2022282090054@whu.edu.cn (J.L.); 2 Institute for Environmental Design and Engineering, The Bartlett, University College London, London WC1H 0NN, UK; j.kang@ucl.ac.uk; 3 Heilongjiang Cold Region Architectural Science Key Laboratory, School of Architecture, Harbin Institute of Technology, Harbin 150006, China; 4 Boxwood A.D.R. Ltd., 2 Orchard Lane, Sheffield S1 2FG, UK; mingkateyang@163.com

**Keywords:** acoustic perception, anxiety, university students, mental health, lockdown

## Abstract

Anxiety significantly impacts the well-being of university students. This study employs the pandemic-induced lockdown as a quasi-experimental situation to examine university students’ perceptions of the acoustic environment and anxiety levels, further investigating the potential correlation between these two variables. An online questionnaire survey was conducted with 250 respondents from seven cities, across nine universities in China, encompassing both high- and low-risk areas concerning the pandemic. In addition, acoustic assessments at three selected sites on the campus were conducted. The results indicate that respondents reported an increase in the perception of indoor noise, particularly voices and instrumental sounds. Concurrently, the sound pressure levels during lockdown were generally lower, resulting in a quieter outdoor acoustic environment. A total of 54% of the respondents reported experiencing anxiety symptoms during this period, and those perceiving changes in the acoustic environment exhibiting higher levels of anxiety. A significant correlation was observed between the acoustic perception and anxiety levels. Overall acoustic satisfaction was negatively correlated with anxiety levels, with this correlation being more pronounced in groups prone to anxiety, such as women. Furthermore, the loudness of the most prominently perceived sound was positively correlated with anxiety levels, with this correlation being stronger in groups less prone to anxiety, such as men. Respondents showed a high level of tolerance for social/communal sounds, voices and instrumental sounds. Conversely, natural and electromechanical sounds were correlated with higher anxiety levels. These findings provide valuable insights for mitigating anxiety through the manipulation of the acoustic environment.

## 1. Introduction

Anxiety disorders are among the most prevalent mental health conditions, characterised by feelings of worry, fear, or unease. These conditions pose a substantial challenge to public health (Penninx et al., 2021; Santomauro et al., 2021). The pervasiveness of anxiety disorders is underscored by their high prevalence (Baxter et al., 2014). Anxiety is particularly prevalent in many countries. Reports indicate that anxiety is the most prevalent mental health disorder in China, affecting an estimated 40 million individuals annually (Y. Huang et al., 2019). Furthermore, the impact of anxiety is far-reaching and often negatively influences physical health, quality of life, and academic performance (Chang et al., 2021). Additionally, the chronic nature of anxiety disorders indicates a persistent condition with the potential for lifelong morbidity (Steel et al., 2014).

Anxiety is especially prevalent among university students (Tan et al., 2023). Given their similar age ranges and the challenges they face, including academic pressure and a critical phase of personal development, university students share similar living environments and comply with unified management. The lockdown conditions during the pandemic period further intensified their collective anxiety levels (Chen et al., 2023). For instance, an online survey in the USA showed that 48.1% of university students reported increased depression or anxiety. Similarly, surveys among French university students reported similar results, with 43% and 39% reporting depression and anxiety, respectively (Essadek & Rabeyron, 2020; X. Wang et al., 2020). Depressive symptoms were also reported by 37% of German university students during the pandemic (Kohls et al., 2021), and similar trends were noted in undergraduate students at UK universities, with a significant rise reaching one-third of the sample in depression and a reduction in well-being during lockdown (Catling et al., 2022). This was particularly evident in China, as the campus lockdown policy resulted in university students spending prolonged periods in high-density and low-privacy spaces, both on campus and in dormitories. These conditions differed significantly from homestays and increased the vulnerability of students who lacked privacy and the companionship of family members during the pandemic. An online survey using the Generalised Anxiety Disorder 7-item scale (GAD-7) among university students in China showed that two-fifths of the sample experienced anxiety symptoms during the lockdown. Additionally, some respondents exhibited persistent or delayed symptoms (Fu et al., 2021).

Anxiety is associated with various factors, among which the acoustic environment plays a crucial role (Ge et al., 2023). Compared to visual stimuli, which can affect anxiety, the influence of sound is considerably more pronounced, with sounds having 4.67 times the impact on anxiety compared to visual stimuli (Jiang et al., 2021; Xu et al., 2024). The relationship between the acoustic environment and anxiety encompasses both positive and negative dimensions. In the positive aspect, the acoustic environment can effectively relieve anxiety. Particularly, the beneficial effects of natural sounds on psychological health have been confirmed (Torresin et al., 2022). A meta-analysis of exposure to natural sounds determined that exposure can markedly reduce anxiety and has the potential to improve cognitive restoration (Zhu et al., 2024). Specifically, birdsong has been shown to have an anxiety-reducing effect (Stobbe et al., 2022). These findings are not limited to green spaces; the efficacy of natural sounds in alleviating anxiety within a medical setting has also been recognised (Watts et al., 2016). Additionally, music combined with treatment can also improve anxiety (O’steen et al., 2021). These observations indicate that the acoustic environment plays a critical role in the regulation of mental states.

While the acoustic environment was found to possess anxiety-alleviating properties, it can also be detrimental to mental health. Particularly, noise contributes to various adverse mental health outcomes (Basner et al., 2014). The perception of annoyance due to noise can lead to poor mental health, negative emotional responses, and elevated levels of perceived stress (Jensen et al., 2018). Higher noise levels are closely associated with increased anxiety, and anthropogenic noise is particularly prone to induce anxiety (Yang et al., 2023; T. Zhou et al., 2020). The association between various noise sources and mental health varies. Specifically, mechanical sounds were found to exhibit a correlation with adverse emotions (Y. Zhou et al., 2022) and there is a positive correlation between traffic noise and the degree of anxiety (Lan et al., 2020). Furthermore, indoor noise has detrimental effects on mental health. Specifically, mental health is positively correlated with annoyances caused by neighbor noise (Persson et al., 2007). A survey conducted by Şentop Dümen et al. (Şentop Dümen & Şaher, 2020) in Turkey confirmed a positive correlation between stress, anxiety levels, noise annoyance, and concerns related to being heard.

Previous studies have extensively investigated the correlation between acoustic perception and anxiety elicited by various stimuli in diverse demographics, including medical treatment, neighborhood activities, and work noise sources. However, unlike the usual anxiety-inducing factors, pandemic-induced anxiety was unprecedented in its scope and impact and triggered a common societal anxiety that was shared universally among all demographics. And the lockdown provided a unique opportunity to study this relationship as they constituted a quasi-experimental situation for both the acoustic environment and anxiety (Alías-Pujol et al., 2024; Kiviruusu et al., 2023; Trudeau et al., 2023). Therefore, this study aimed to explore the correlation between acoustic perception and anxiety during the pandemic-induced lockdown. The research sample consisted of university students, and the context was a university campus with a high population density. The research questions addressed in this study were as follows: (1) How did university students perceive the acoustic environment during the pandemic-induced lockdown? (2) What were their anxiety levels during the lockdown? (3) What were the correlations between acoustic perception and anxiety levels?

## 2. Materials and Methods

### 2.1. Methodology

**Figure 1:**
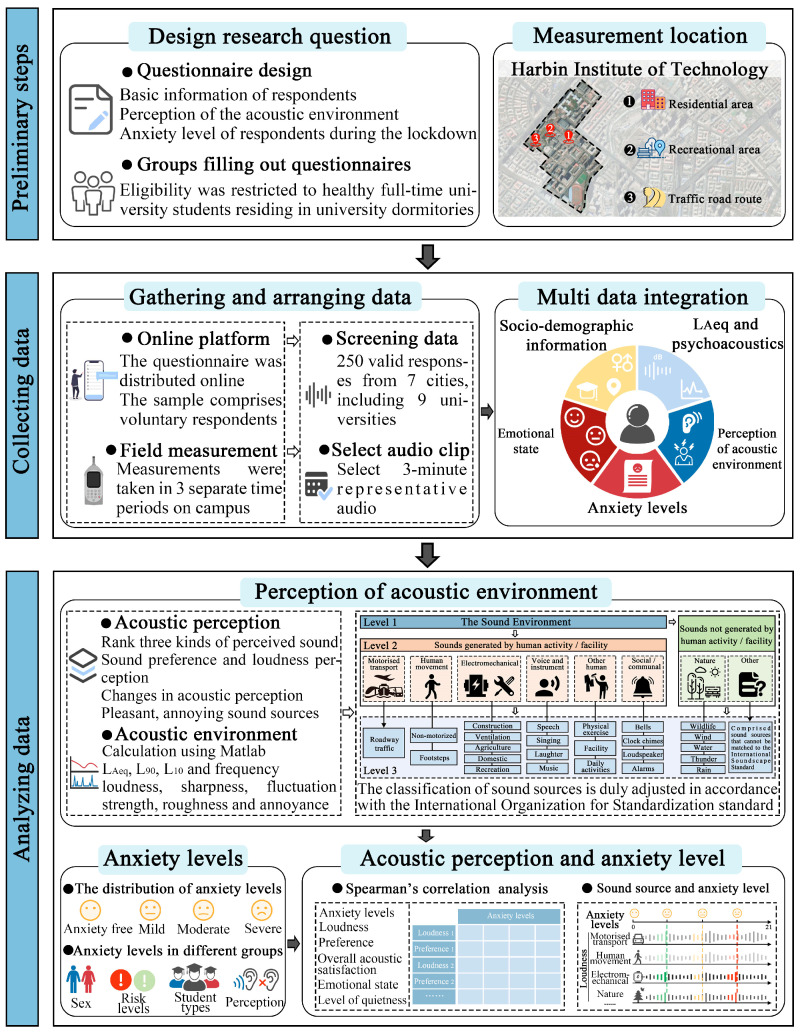
Research framework.

In this study, the research methodology was centered on questionnaire surveys, on-site field measurements, and data analysis (Figure 1). The research framework was as follows: (1) preliminary steps: designing the research questions, identifying the target respondents, and selecting the measurement locations; (2) collecting data: an online questionnaire survey was conducted among university students to gather data on their acoustic perception and anxiety levels. Meanwhile, on-site acoustic signal collection was carried out at diverse and representative locations to ensure sample diversity; (3) analysing data: sound source classification for acoustic environment perception and the distribution of anxiety levels were analysed. Finally, the relationship between acoustic perception and anxiety levels was examined.

### 2.2. Questionnaire Survey

A questionnaire was used to collect data on the acoustic perceptions and anxiety levels. The questionnaire was divided into four sections, containing a total of 37 questions. The first section collected the subjects’ demographics, including sex, student type, and address, for subsequent comparative analysis. The second section assessed the mental state using the GAD-7. The GAD-7 is a validated instrument for screening and assessing the severity of generalised anxiety disorder (Spitzer et al., 2006); it was confirmed as a reliable and effective self-report measure for general anxiety disorder and has been effectively applied in previous domestic research (M. Wang et al., 2021). The GAD-7 is a seven-item, one-dimensional scale where respondents rate their anxiety levels on a 4-point Likert scale (0 = Not at all to 3 = Nearly every day). The total score is calculated by summing all item responses, ranging from 0 to 21, with higher total scores indicating greater anxiety levels (Krizova et al., 2024). GAD-7 scores ranging from 0 to 4, 5 to 9, 10 to 14, and 15 to 21 represent anxiety free, mild, moderate, and severe anxiety, respectively (F. Wang et al., 2024). Therefore, the GAD-7 was used in this study to evaluate the anxiety levels of respondents. The third section focused on the acoustic perception of the respondents. To investigate the correlation among perceived sound source types, overall acoustic perception and anxiety levels, respondents in the study were required to undergo a comprehensive evaluation procedure. Initially, respondents were instructed to rank the three most noticeable sounds (X. Zhang et al., 2018), with the option to provide supplementary descriptions or examples if deemed necessary. Subsequently, the respondents evaluated the ratings of preferences and perceived loudness for each sound using a five-point Likert scale (1–5) (Hamida et al., 2023). Then, the respondents utilized a free-text format to elaborate on the variations in their acoustic perceptions, including the sound sources they perceived as having increased or decreased, and those they desired to hear the most. Next, a five-level Likert scale (1–5 points) was employed to evaluate their overall acoustic satisfaction, level of quietness within the acoustic environment, and their emotional state. Finally, respondents were asked to identify their pleasant and annoying sound sources and to rate their preference for these sounds on a scale of 1–5. Additionally, to identify other potential factors associated with anxiety, the fourth section explored the multifaceted impact of the lockdown on individuals. Respondents were instructed to select the factors that most influenced them during the lockdown and assess their level of fear of COVID-19 on a scale of 1 to 5. Subsequently, open-ended questions were utilized to clarify the pandemic’s effects on their travel, acoustic environment, virtual experiences, and other relevant aspects.

The survey utilized distinct question types, including (a) open questions that do not provide answer options and solicit responses from respondents; (b) single-choice questions that constrain respondents to selecting one answer option; and (c) Likert scales that consist of a rating scale of 5 levels. The types of questions pertaining to the questionnaire are available in the Supplementary Material.

The questionnaire survey was designed and conducted in accordance with the general principles of the Declaration of Helsinki (World Medical Association, 2013). Prior to participation, each respondent was given information about the purpose and procedures of the study, the confidentiality of their responses, and the voluntary nature of their participation. The survey was anonymous, the respondents confirmed that they were 18 years of age or older. Consent was then confirmed using checkboxes. Data were presented anonymously.

The data collection and management for this study were facilitated through the online platform “survey star” (powered by www.wjx.com). The efficacy of internet-based surveys has been substantiated in prior research (F. Liu et al., 2022). The questionnaire was accessible via web browsers from April 10th to 24th 2022 and shared via social networks. Consequently, the sample comprises voluntary respondents rather than being derived from non-probability methods such as random sampling. Eligibility was restricted to healthy full-time university students residing in university dormitories, yielding a total of 269 completed questionnaires. Based on the completion of the questionnaire and to achieve a balanced sample size across various cities, only 250 valid responses were retained for analysis. The validity of this sample size has been confirmed in previous studies (X. Zhang et al., 2018). The demographic distribution was relatively balanced, comprising 51.6% women and 48.4% men among the respondents. The questionnaires were collected from 7 cities nationwide, covering 9 universities. Based on the pandemic situation at the time of questionnaire submission, respondents were categorised into high-risk and low-risk areas. Among them, 157 (62.8%) were from high-risk areas and 93 (37.2%) were from low-risk areas. Furthermore, the address information provided by the respondents indicated that the Harbin Institute of Technology contributed the largest sample size, representing 49.6% of the total questionnaires collected.

### 2.3. Field Measurements

**Figure 2:**
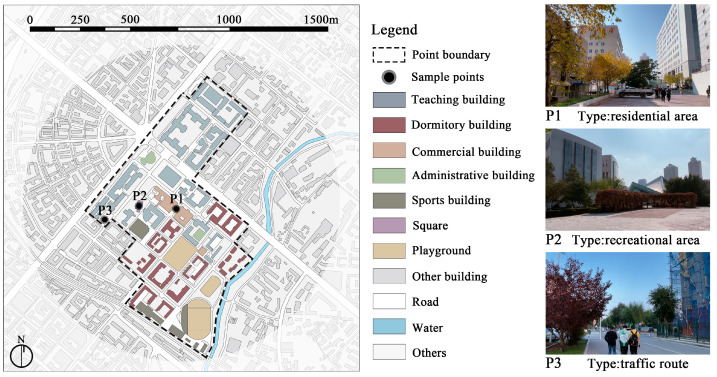
Acoustic environment acquisition locations.

The field measurement location was selected based on the expected region with the highest anticipated survey response rate, ensuring a robust dataset for meaningful analysis. Given the substantial student population and extensive research engagement at Harbin Institute of Technology in China, we chose this institution as the site for our field measurement. Due to the pandemic-induced centralized management within the campus and restrictions on indoor gatherings, this research concentrated on the assessment of outdoor acoustic environment. Acoustic environmental samples were collected from various locations within the campus during and after the lockdown period (Figure 2). To capture the acoustic characteristics of different spatial types, the measurement locations included a residential area (P1), recreational area (P2), and traffic routes (P3). With the exception of P3 which is interface with the off-campus environment, the other locations are open spaces within the campus area. Specifically, P1 is positioned at the entrance of a supermarket in the residential area, surrounded by educational buildings, student dormitories, and canteens; it serves as the primary daily living space for students on campus. P2 is situated in a square adjacent to the university kindergarten, which serves as an open space for leisure activities. P3 is situated near the campus’s main entrance and exit, adjacent to a parking lot and an off-campus traffic road.

The measurement locations were dispersed throughout various regions of the campus to avoid interference among them. The measurements were conducted using a sound level meter (AWA6228+). The collection of acoustic data was carried out during the lockdown (6 April 2022) and after the lockdown (13 October 2023). To ensure data comparability, both measurements were completed at the same fixed locations, including three distinct time intervals: morning (8:00 AM), noon (12:00 PM), and night (8:00 PM), with each measurement spanning five minutes. The measurement range covered both low-range (20–132 dBA) and high-range (30–142 dBA). Additionally, to ensure that the measurements were not affected by external disturbances that could cause data errors, the instruments were positioned 5 m from the edges of the activity areas (Eriksson, 1991). Meanwhile, a bracket system was used to place the instrument at a height of 1.5 m above ground level during the measurement to obtain a high-fidelity stereo recording (F. Liu & Kang, 2018).

### 2.4. Data Analysis

In this study, data analysis was carried out using SPSS 27.0, employing a series of steps to process and analyse the data obtained from both questionnaire surveys and field measurements. The acoustic perception evaluations were ranked data, whereas the anxiety levels were quantified as metric data. A series of analyses were conducted, including reliability testing, nonparametric tests, and Spearman’s correlation analysis. First, the reliability of the scale was assessed by assessing the consistency and reliability of the questionnaire responses. Cronbach’s alpha coefficients ranged from 0.633 to 0.923, indicating that the questionnaire responses were sufficiently reliable for further statistical analyses. Second, to characterize the distribution of different sound source types in the acoustic perception, this study employed the 2018 International Soundscape Standard (ISO/TS 12913-2) (ISO, 2018) as a reference for sound classification. However, due to the partial suitability of the ISO classification for our specific study, adjustments were made to better align with the context of this research. Specifically, the category of domesticated animals was incorporated into the natural classification, and a new category designated “other” was established to encompass sound sources not addressed in the ISO classifications. Consequently, the primary classifications include motorised transport, human movement, electromechanical, voice and instrumental, other humans, social/communal, natural, and others. Descriptive statistical analyses were used to assess the proportion of each sound source type in the acoustic perceptions reported. Additionally, descriptive analysis was conducted to examine anxiety levels across different demographic groups. Subsequently, a nonparametric test was used to analyse whether there were significant differences in anxiety levels among different demographics, where the mean values were primarily utilized to represent the anxiety levels in this study. In the final step, Spearman’s correlation analysis was utilized to examine the correlation between acoustic perception and anxiety levels. For open-ended questions, the responses were classified into positive and negative categories. This classification was based on salient terms that were carefully extracted from the respondents’ responses.

Additionally, the sound files were equalised according to the measured SPL. Initially, three measurements were taken at each location in the morning, noon, and night periods respectively, with each measurement comprising 5 min of audio sampling. Subsequently, a continuous three-minute segment was extracted from each audio sample and processed using the Matlab. For each sound sample, a sequence of instantaneous psychoacoustic parameters and the A-weighted equivalent continuous sound pressure level (*L*_Aeq_) and frequency were derived, reflecting human auditory sensations (Hamida et al., 2024), and the average values for each parameter were calculated. The psychoacoustical parameters include loudness (sone), sharpness (acum), fluctuation strength (vacil), and roughness (asper). Among them, loudness describes the subjective perception of absolute and relative sound intensities, reflecting the direct response of listeners to the sound pressure level; sharpness quantifies the proportion of high-frequency components within the sound spectrum, and the higher the proportion of high-frequency components in a sound signal, the higher the sharpness value; fluctuation strength characterizes the magnitude of instantaneous changes in a sound signal, with a primary focus on low-frequency modulated signals and roughness refers to the perception of instantaneous fluctuations in high-frequency sound signals, and rough sounds typically evoke an unpleasant auditory sensation (ISO, 2017; J. Kang, 2006). Meanwhile, Equations (1)–(3) of Zwicker’s model were utilized to compute the psychoacoustic annoyance (*PA*) based on loudness, sharpness, fluctuation strength and roughness, which reflects the degree to which noise is perceived as annoying (Hartmann, 2001; Nowakowski & Komorski, 2024).

(1) $$PA=N_{5}\left(1+\sqrt{w_{S}^{2}+w_{FR}^{2}}\right)$$

(2) $$w_{S}=\frac{\left(S-1.75\right)\cdot \log\left(N_{5}+10\right)}{4}$$

(3) $$w_{FR}=\frac{2.18\cdot \left(0.4F+0.6R\right)}{N_{5}^{0.4}}$$

where *PA* represents psychoacoustic annoyance; *N*_5_ represents the fifth percentile of the loudness; $w_{S}$ quantifies the impact of sharpness *S*; and $w_{FR}$ represents the effect of fluctuation strength *F* and roughness *R* (Di et al., 2016).

## 3. Results

### 3.1. Characteristics of Acoustic Perception and Acoustic Environment

#### 3.1.1. People’s Perception of the Acoustic Environment

**Figure 3:**
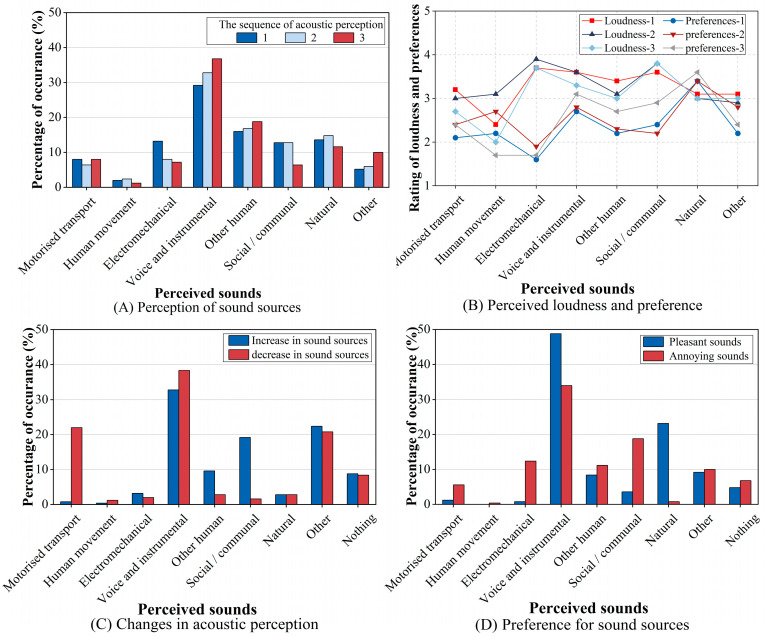
Composition of acoustic environment perception. (**A**) Ranking of the three most noticeable sound sources. (**B**) Perceived loudness and preference (1 = strongly small/dislike; 5 = very loud/like). The numbers after loudness and preference represent the order of perception. (**C**) Variation in perception of the acoustic environment compared to the pre-lockdown. (**D**) The pleasant and annoying sound sources.

The multi-dimensional acoustic perception evaluation values are illustrated in Figure 3. Figure 3A,B indicate that the dominance of voice and instrumental sounds is evident in terms of perceived occurrence, preference, and loudness. In contrast, natural sounds demonstrated lower perceived occurrences and loudness but received the highest preference ratings. Conversely, social/communal and electromechanical sounds showed relatively high loudness but exhibited lower perceived occurrences and preferences. Figure 3C shows that most respondents noticed changes in sound sources, with only 22.4% reporting no increase and 20.8% reporting no decrease. Specifically, among the increase in sound sources perceived by the respondents, voice and instrumental sounds (32.8%) were the most frequent occurrences, primarily originating from indoor activities, followed by social/communal sounds (19.2%), mostly from loudspeakers. For decreased sound sources, voice and instrumental sounds (38.4%) were again the most frequent, primarily stemming from outdoor daily activities, followed by the less preferred sounds of motorised transport (22.0%). Additionally, 34.0% and 18.8% of the respondents reported that the perceived increases in voice and instrumental sounds and social/communal sounds, respectively, caused annoyance (Figure 3D).

These results suggest that voice and instrumental sounds were more frequently perceived by the respondents. However, the perceived frequency of occurrence of most sound sources did not consistently align with the perceived loudness and preference levels. Furthermore, the respondents’ perceptions of the acoustic environment shifted during the lockdown period. Specifically, the decreased perception of motorised transport sounds had a positive effect, while the increased perception of voice and instrumental, as well as social/communal sounds, caused annoyance among most respondents.

**Figure 4:**
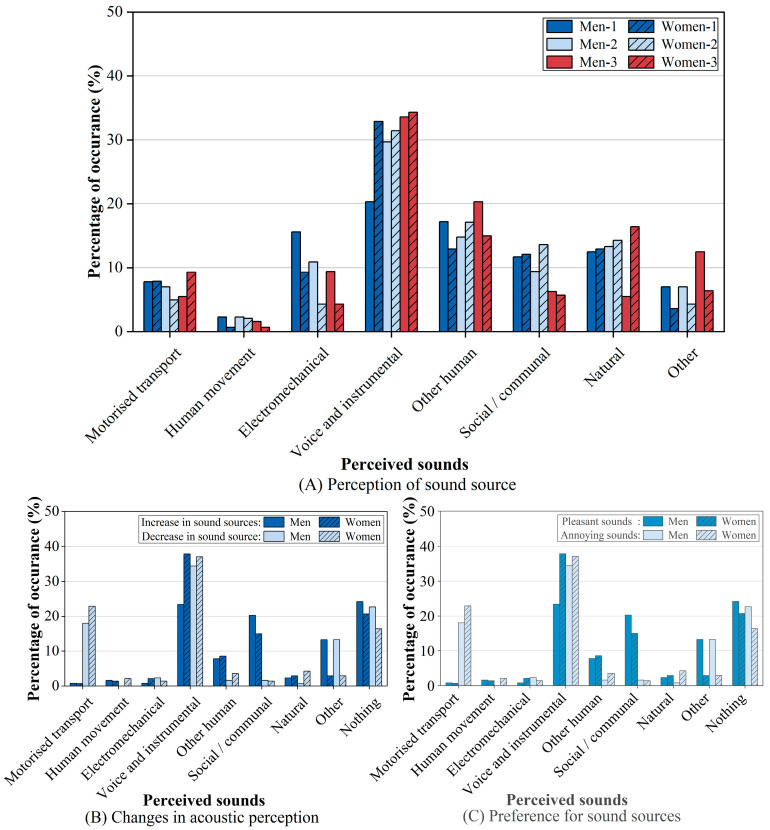
Acoustic environment perception differences according to sex. (**A**) Sex differences in acoustic perception (The numbers after sex represent the order of acoustic perception). (**B**) Sex differences in perception of acoustic environment variation. (**C**) Sex differences in the pleasant and annoying sound sources.

Furthermore, sex differences in acoustic perception and sound preferences were analysed (Figure 4). As shown in Figure 4A, women reported higher perceived frequencies for natural sounds, voice and instrumental sounds compared to men, while men were more sensitive to electromechanical sounds. Additionally, the percentage of women who perceive changes in acoustic perception is 9.8% higher than that of men (Figure 4B). Regarding pleasant sounds (Figure 4C), both sexes indicated a higher pleasantness for voice and instrumental sounds, but men showed a higher pleasantness towards music sounds within this category, while women favored speech sounds. For annoying sounds (Figure 4C), a higher proportion of women (21.4%) expressed aversion to social/communal sounds compared to men (12.5%).

The results suggest that women were more sensitive to natural, voice and instrumental sounds and have a more pronounced ability to perceive variations in the acoustic environment. In addition to differences in acoustic perception, there were also differences in sound preferences between the sexes. Specifically, men prefer musical sounds, whereas women prefer speech sounds.

#### 3.1.2. Variations in On-Site Acoustic Environment Measurement

**Figure 5:**
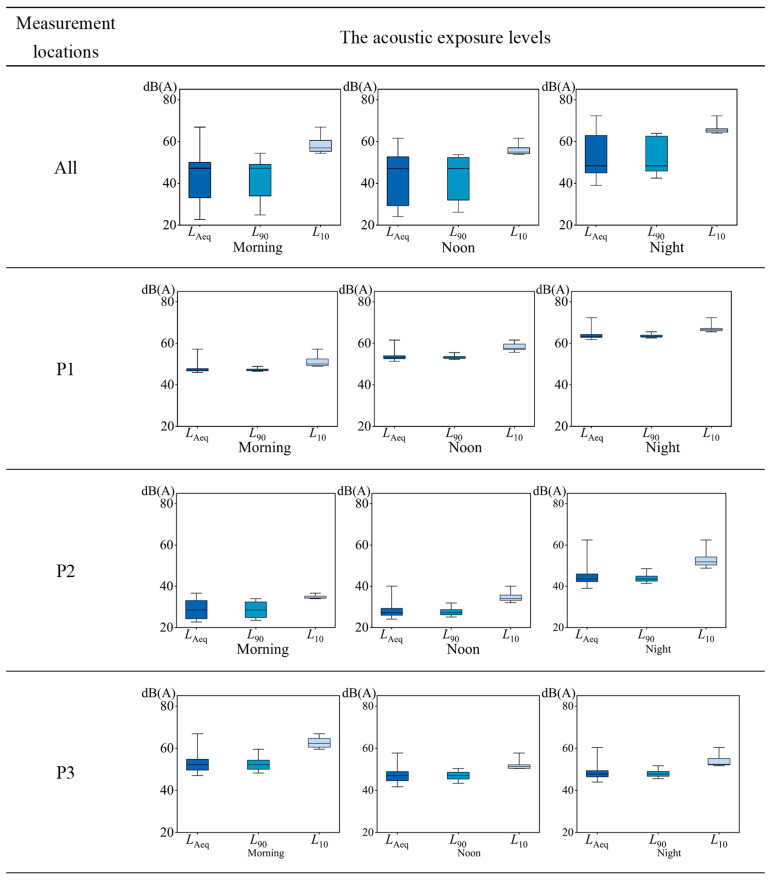
Box plot of exposure levels (*L*_Aeq_, *L*_90_, *L*_10_) at measurement locations during the lockdown, with error bars indicating the maximum–minimum range and box limits representing the 25th and 75th percentiles.

In terms of the overall acoustic exposure level (Table 1), the SPL exhibited higher fluctuations throughout the day and remained mostly lower during the lockdown compared with regular time after the lockdown. Specifically, the results indicate a decrease in daytime sound pressure levels by 12.9 to 14.4 dB during the lockdown, the highest difference in *L*_Aeq_ was observed at noontime. In contrast, nighttime differences were relatively negligible. Furthermore, the *L*_Aeq_ difference between the two periods was the largest at P2, while the difference at P1 was relatively smaller. Additionally, there were variations in acoustic exposure levels between different locations during the lockdown (Figure 5). The average *L*_Aeq_ at the residential area (P1) and the traffic route (P3) was around 50 dBA, higher than that at the recreational area (P2), which ranged from 28.7 to 44.5 dBA. Similarly, the average *L*_90_ and *L*_10_ values at P1 and P3 were also higher compared to those at P2.

The results reveal that, generally, human activities were significantly reduced during the lockdown period, resulting in lower acoustic exposure levels. This reduction was particularly evident in recreational areas, where human activities were more restricted compared to residential areas and traffic routes.

**Figure 6:**
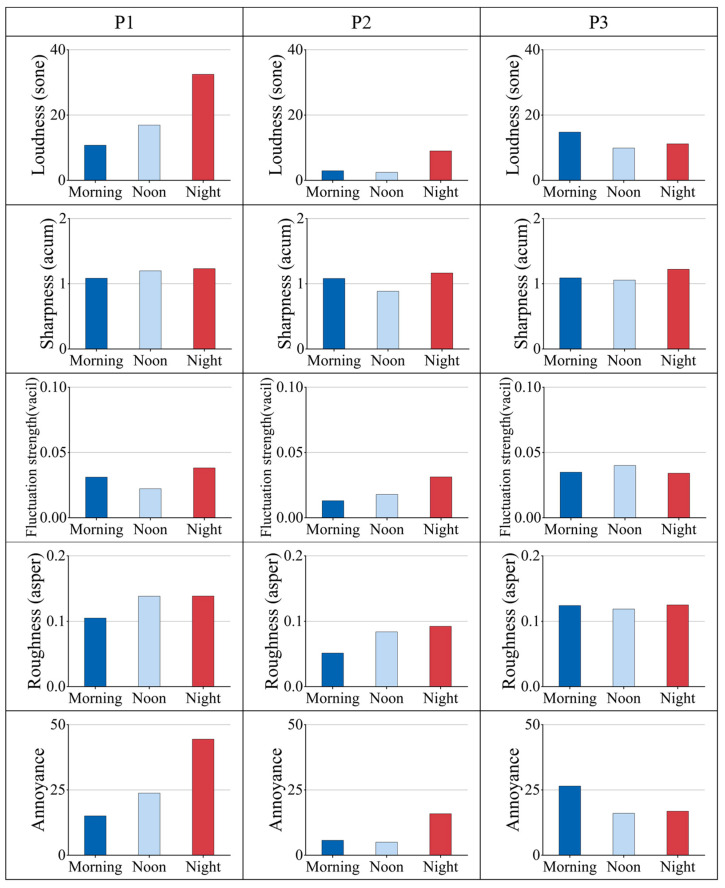
Mean values of psychoacoustic parameters during the lockdown.

Psychoacoustic evaluation revealed distinct differences in four parameters across the measurement locations: loudness, sharpness, fluctuation strength, and roughness (Figure 6). Regarding loudness, P1 exhibited a notably higher level compared to P2 and P3, averaging between 10.74 and 32.46 sone. In terms of sharpness, the differences among the three measuring points were minimal, with mean differences between 0.01 and 0.90 acum. In terms of fluctuation strength, the overall fluctuation strength at P3 is relatively high. In contrast, P2 generally had lower levels, averaging between 0.01 and 0.03 vacil but P1 and P2 experienced increased fluctuation strength during nighttime. Similarly, roughness was highest at P1, averaging between 0.10 and 0.14 asper, while P2 showed lower values. In terms of *PA*, the findings indicated that P2 exhibited the lowest *PA* values, ranging from 5.7 to 15.9. Conversely, P1 demonstrated higher *PA* values, particularly during nighttime, with an average of 44.4. P3 showed the highest average *PA* value of 26.5 in the morning.

**Figure 7:**
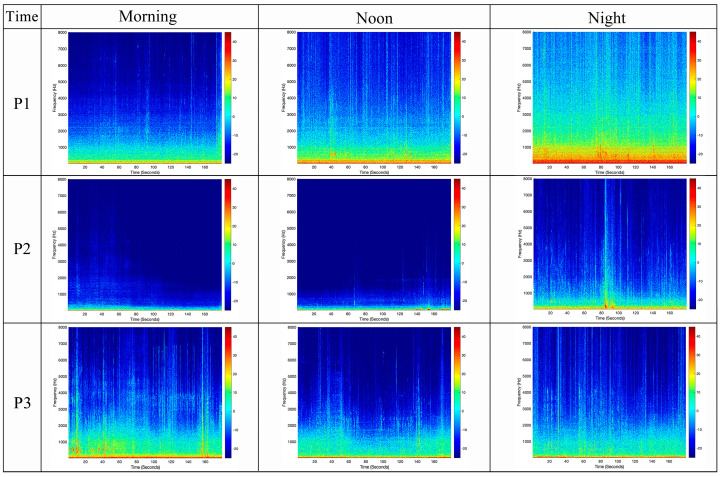
Time–frequency spectrograms of each selected measuring location during the lockdown, presenting continuous three-minute segments within a frequency range of 0–8000 Hz.

This result corresponds to the spectrogram shown in Figure 7. Despite the three measurement locations primarily consisting of low-frequency components, P1 has a broader frequency range compared to P2 and P3, with both its high-frequency and low-frequency components significantly higher. Additionally, P1 demonstrates higher sound energy within the 0–1 kHz range. P2 is primarily centered within the 0–500 Hz range, exhibiting relatively small fluctuations in amplitude within its low-frequency band and a relatively uniform sound energy distribution during daytime. However, during nighttime, the sound energy within the low-frequency band of P2 is significantly higher. In contrast, P3 exhibits noticeable fluctuations in amplitude within the 0–2 kHz range.

Overall, the results suggest that the lockdown affected the acoustic environment of different types of spaces. The acoustic environment became quieter and more stable, particularly during daytime. However, the acoustic environment demonstrated a relatively active state at nighttime. It is noteworthy that the acoustic environment of the recreational area and the traffic route was more tranquil and stable, while the acoustic environment in residential areas was relatively active, exhibiting the highest level of annoyance. In contrast, leisure areas had the lowest level of annoyance.

### 3.2. Respondents’ Anxiety Levels

**Figure 8:**
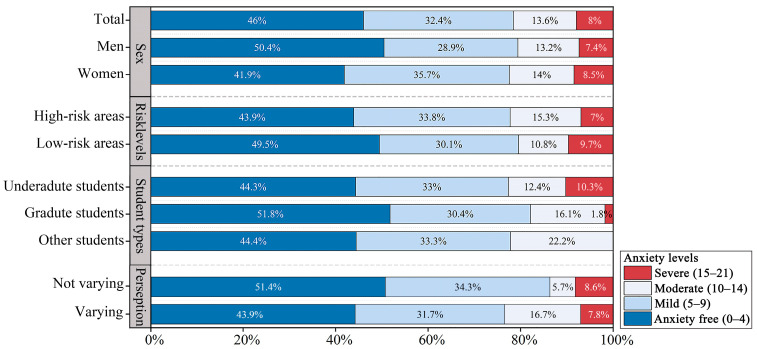
Distribution of respondents’ anxiety levels.

The mental state assessment exhibits that anxiety symptoms were relatively prevalent among the surveyed population, with 54% of respondents exhibiting varying degrees of anxiety, categorised as mild (32.4%), moderate (13.6%), or severe (8%) anxiety, respectively (Figure 8). Among different demographic groups, women (58.1%), undergraduates (55.7%), and students from high-risk areas (56.1%) were more prone to exhibiting anxiety symptoms compared to men (49.6%), graduate students (48.2%), and students from low-risk areas (50.5%), respectively. These trends were particularly pronounced within the mild anxiety group. Meanwhile, the proportion of anxiety was observed to be 7.5% higher among respondents who perceived changes in the acoustic environment than among those who did not perceive such variations.

**Figure 9:**
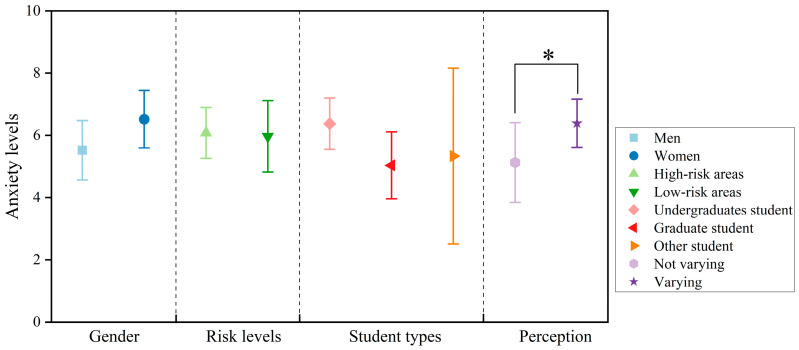
The mean values of anxiety levels of respondents from different groups (Error bars: 95% CI). * represents *p* < 0.05.

However, as illustrated in Figure 9, despite women (mean = 6.5) exhibiting a higher anxiety level than men (mean = 5.5), no statistically significant differences in anxiety levels were observed among groups classified according to sex, student type, and risk level of areas (*p* = 0.06 > 0.05). Moreover, the respondents who perceived variations in the acoustic environment (mean = 6.4) exhibited significantly higher anxiety levels than those who did not perceive such variations (mean = 5.1), with a significant difference in anxiety levels between the two groups (*p* = 0.03 < 0.05).

The results reveal that more than half of the student population experienced anxiety during the lockdown period. Among the different groups, those who were more sensitive to variations in the acoustic environment tended to experience higher anxiety levels and were more prone to anxiety symptoms. Moreover, women, undergraduates, and students from high-risk areas exhibited a higher proportion of anxiety, and these groups were more prone to anxiety.

### 3.3. The Relationship Between Anxiety Levels and Acoustic Perception

#### 3.3.1. The Relationship Between Overall Anxiety Levels and Acoustic Perception

**Figure 10:**
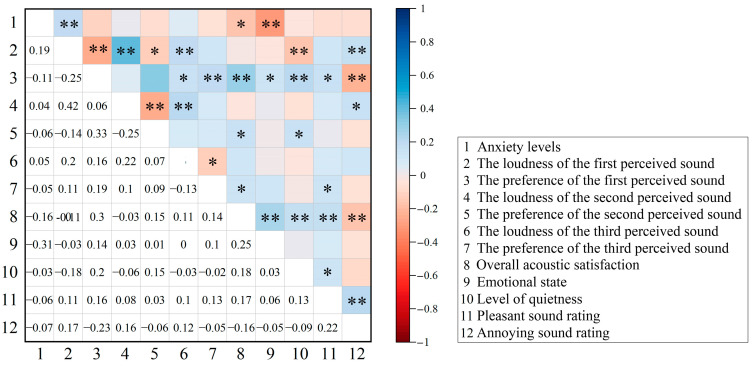
The correlation between the anxiety levels and acoustic perception, overall acoustic satisfaction, level of quietness and emotional state, including the two-tailed significance levels. Significant correlations are marked with * (*p* < 0.05) and ** (*p* < 0.01).

The correlation between acoustic perception and anxiety was explored using Spearman’s analysis (Figure 10). The results indicated a positive correlation between anxiety levels and the loudness of the first perceived sound (*r* = 0.19, *p* < 0.01). Conversely, there was a negative correlation between anxiety levels and overall acoustic satisfaction (*r* = −0.16, *p* < 0.05), and emotional state (*r* = −0.31, *p* < 0.01).

**Figure 11:**
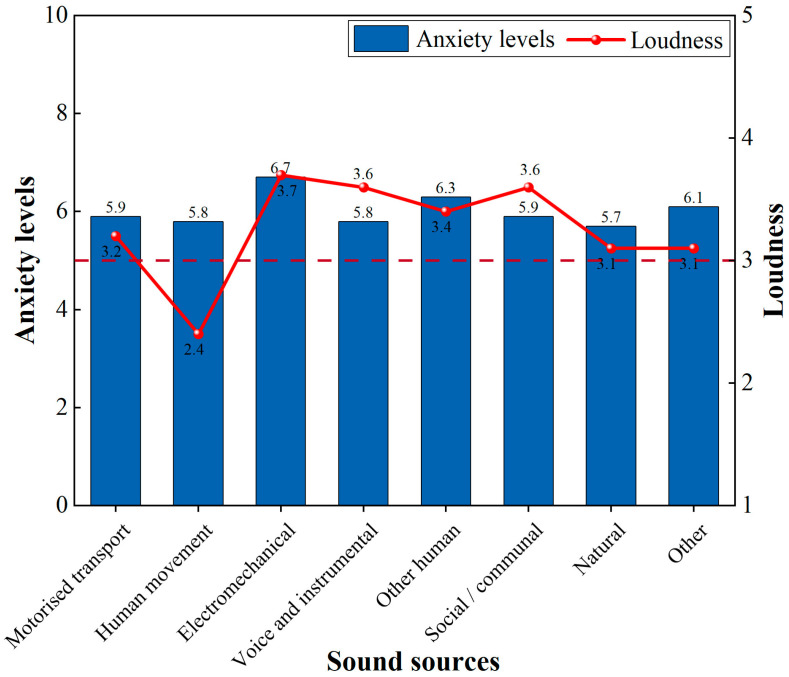
Anxiety levels and the loudness of the first perceived sound (Anxiety levels: 0–4 = anxiety free, 5–9 = mild, 10–14 = moderate, 15–21 = severe; the loudness is graded as per the following linear scale: 1 = strongly small; 2 = small; 3 = neutral; 4 = loud; and 5 = very loud). The horizontal dashed line represents the threshold for presence of anxiety symptoms.

Furthermore, we analysed the correspondence between anxiety levels and the loudness of the first perceived sound source (Figure 11). The perception of loudness for electromechanical sounds was high, with respondents reporting the highest anxiety levels (6.7). In contrast, the perceived loudness of natural sounds by respondents was low, correlating with the lowest anxiety levels (5.7). However, the perceived loudness was relatively high for voice and instrumental (5.8), and social/communal sounds (5.9), but these types of sounds corresponded to the lower anxiety levels.

Further correlation analysis explored specific relationships between different sound sources and anxiety levels (Table 2). There was a positive correlation between anxiety levels and electromechanical (*r* = 0.467, *p* < 0.01) and natural sounds (*r* = 0.339, *p* < 0.05), with the most significant correlation observed for electromechanical sounds.

All these results suggest that respondents’ anxiety levels increased when increasing the loudness of the first perceived sound. Specifically, as the perceived loudness of the electromechanical and natural sounds increased, the respondents’ reported anxiety levels also increased accordingly. However, the higher tolerance of respondents to voice and instrumental sounds, social/communal sounds appeared to weaken the correlation between anxiety levels and perceived loudness. Conversely, as overall acoustic satisfaction and emotional state improved, anxiety levels decreased.

#### 3.3.2. The Relationship Between Anxiety Levels and Acoustic Perception Based on Different Demographics

**Figure 12:**
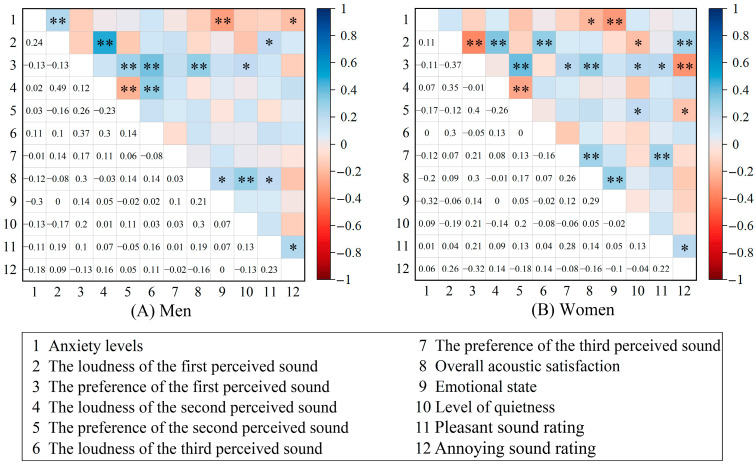
The correlation between the anxiety levels and acoustic perception, overall acoustic satisfaction, level of quietness, emotional state according to the sexes, including the two-tailed significance levels. Significant correlations are marked with * (*p* < 0.05) and ** (*p* < 0.01).

The correlation analysis indicated (Figure 12) that anxiety levels for both men (*r* = −0.30, *p* < 0.01) and women (*r* = −0.32, *p* < 0.01) were significantly negatively correlated with emotional state, representing the most pronounced correlation among the factors examined, indicating that anxiety levels increased with a decrease in emotional state. In terms of acoustic perception, the anxiety levels of men were positively correlated with the loudness of the first perceived sound (*r* = 0.24, *p* < 0.01), whereas for women, anxiety levels were negatively correlated with overall acoustic satisfaction (r = −0.20, *p* < 0.05).

**Figure 13:**
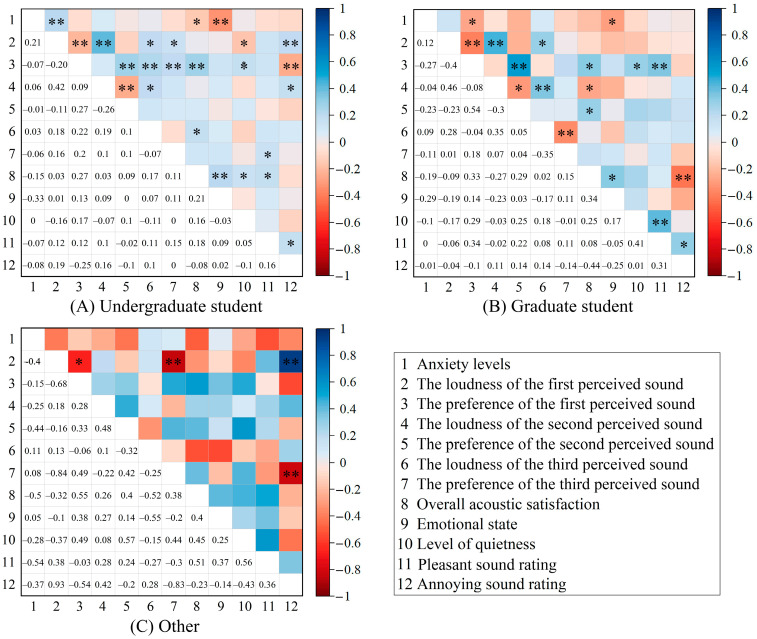
The correlation between the anxiety levels and acoustic perception, overall acoustic satisfaction, level of quietness, emotional state by different student types, including the two-tailed significance levels. Significant correlations are marked with * (*p* < 0.05) and ** (*p* < 0.01).

Furthermore, this study analysed the differences in the correlation between anxiety levels and acoustic perception among various student types (Figure 13). Graduate students exhibited a negative correlation between anxiety levels and preference for the first perceived sound (*r* = −0.27, *p* < 0.05), and emotional state (*r* = −0.29, *p* < 0.05). Conversely, the anxiety levels of undergraduate students were not only negatively correlated with emotional state (*r* = −0.33, *p* < 0.01) and overall acoustic satisfaction (*r* = −0.15, *p* < 0.05) but also positively correlated with the loudness of the first perceived sound (*r* = 0.21, *p* < 0.01). However, other student types did not exhibit significant correlations between acoustic perception and anxiety levels (*p* > 0.05).

**Figure 14:**
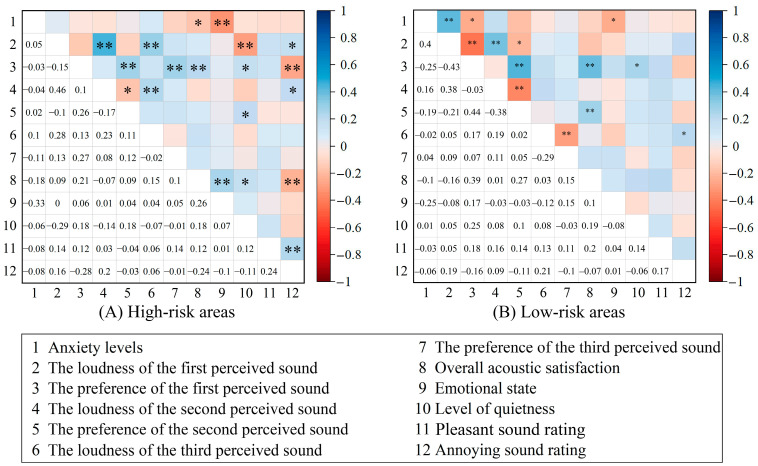
The correlation between the anxiety levels and acoustic perception, overall acoustic satisfaction, level of quietness, emotional state by different risk levels of areas, including the two-tailed significance levels. Significant correlations are marked with * (*p* < 0.05) and ** (*p* < 0.01).

We also examined whether the correlations between acoustic perception and anxiety levels were affected by the different risk levels of the areas (Figure 14). In low-risk areas, anxiety levels were positively correlated with the loudness of the first perceived sound (*r* = 0.40, *p* < 0.01) and negatively correlated with preference for the first perceived sound (*r* = −0.25, *p* < 0.05). In high-risk areas, anxiety levels showed no correlation with the first perceived sound, yet it exhibits a negative correlation with overall acoustic satisfaction (*r* = −0.18, *p* < 0.05) as well as emotional state (*r* = −0.33, *p* < 0.01).

Overall, these results suggest that distinct student demographics exhibited variations in the correlation between acoustic perception and anxiety. Specifically, the anxiety levels of women and students in high-risk areas were negatively correlated with overall acoustic satisfaction, whereas other student groups exhibited a positive correlation between the loudness or preference of the first perceived sound and anxiety levels. The anxiety levels of undergraduate students were related to both acoustic perception factors. Thus, in populations that are more susceptible to anxiety, attention should be paid to the overall satisfaction with the acoustic environment. Conversely, for individuals less prone to anxiety, the first perceived sound is more critical in shaping their mental health. Furthermore, emotional state was associated with anxiety levels in multiple student groups, with the most significant correlation observed in individuals in high-risk areas.

## 4. Discussion

### 4.1. Variations in the Perception of the Acoustic Environment

This study examines alterations in the perception of the acoustic environment during the pandemic-induced lockdown. During this period, the implementation of containment policies restricted people’s behavior, leading to numerous changes in our daily lives. The suspension of various activities and changes in work patterns affected the acoustic environment. Prior research has substantiated a decline in outdoor noise levels during the COVID-19 pandemic compared to regular periods. In London, there was a decrease ranging from 1.2 dB to 10.7 dB, with an average reduction of 5.4 dB across 11 locations (Aletta et al., 2020). Similarly, in Madrid, noise levels diminished by 4 dB to 6 dB during the lockdown period (Asensio et al., 2020). In Dublin, reductions were observed at 11 out of 12 monitoring stations, ranging from 2.8 dB to 6.3 dB (Basu et al., 2021). Meanwhile, in Shizuoka Prefecture in Japan, noise levels in residential areas exhibited minimal changes, with reductions of less than 3 dB during the partial lockdown (Sakagami, 2020). However, there was an increased perception of indoor noise found during this period (Lee & Jeong, 2021). These reductions can largely be attributed to the decrease in traffic and human activity, resulting in noticeably quieter urban environments. The findings of our study are consistent with these observations and further underscore the universality of acoustic environmental modifications during the pandemic.

Furthermore, this study also confirms the existence of sex differences in acoustic perception (Guo et al., 2022). Previous research has shown that women residing in crowded residential buildings are more susceptible to the impact of noise on their well-being than men (Caniato et al., 2021). Similarly, this study found that women were more sensitive to variations in perceptions of the acoustic environment than men, especially concerning natural, voice and instrumental sounds. However, it is noteworthy that women’s perception of natural sounds might decrease during the epidemic (Bartalucci et al., 2022). These differences may stem from variations in the psychological states of subjects across different research contexts, especially as stress and uncertainty during the pandemic may exacerbate sex differences in sound perception. Furthermore, the sample in the study (Bartalucci et al., 2022) was primarily drawn from citizens residing in various living environments, while this study focused on the university student population, a younger and cognitively flexible group that may exhibit different response patterns when facing acoustic environment changes. Meanwhile, university students typically live in relatively centralized and well-regulated campus environments, which to some extent reduces the interference of external environmental factors.

### 4.2. Anxiety Levels in Different Demographics

This section primarily delves into a detailed analysis and discussion of anxiety levels among university students during the lockdown period. The findings of this study reveal that university students experienced varying degrees of anxiety during the lockdown, which aligns with existing research findings (J. Huang & Liu, 2023; Lv et al., 2023). Notably, previous research has already confirmed that sex is a factor influencing anxiety, with women being more susceptible to negative psychological impacts than men (Bai et al., 2025; Santomauro et al., 2021). The results of this study confirm this viewpoint. Sex differences in anxiety levels may stem from multiple factors, including women potentially bearing higher responsibilities during the pandemic, facing greater economic impacts, and experiencing exacerbated stress (Hakami et al., 2021; Wenham et al., 2020). Additionally, undergraduates and students from high-risk areas were found to be more prone to anxiety, which is consistent with previous research (Gottlieb et al., 2024; Y. Zhang et al., 2021). This may be attributed to the fact that undergraduates face more stress from school, such as academic, interpersonal, employment, and economic challenges, and that a higher risk of infection has been associated with an increase in anxiety symptoms (Borgi et al., 2023; Y. Liu et al., 2022). However, there are also studies that contradict this finding, indicating that anxiety is not correlated with the regions they live (C. Wang & Zhao, 2020). These conflicting research results suggest that the relationship between anxiety and geographical location may be complex and influenced by various factors, including individual differences. Consequently, more attention should be directed towards women, undergraduates, and students from high-risk areas when addressing the issue of student mental health. By recognising the susceptibility of these populations, tailored interventions can be devised to bolster their mental health.

### 4.3. The Correlation of Acoustic Perception and Other Factors with Anxiety Levels

Regarding the correlation between acoustic perception and anxiety, prior research has established a link between traffic noise and anxiety (Y. Zhang et al., 2017). However, despite traffic noise being significantly reduced during the pandemic, more than half of the university students surveyed still showed symptoms of anxiety. Moreover, individuals who are more sensitive to alterations in the acoustic environment are not only more susceptible to anxiety but also experience higher levels of it. Similar observations were noted in both residential settings and work environments (Şentop Dümen & Şaher, 2020; Yang et al., 2023). These findings suggest that, although traffic noise is correlated with anxiety, other factors may also be associated with anxiety in the context of the pandemic. For example, in the open-ended responses of this study, 69.4% of respondents indicated that the pandemic increased their online activities, and nearly half of the students expressed an aversion to online learning methods. This could be attributed to the shift in teaching methodologies during the pandemic, which exacerbated the psychological burden on both educators and students, thereby contributing to student anxiety via online learning methods (Abdous, 2019; Curelaru & Curelaru, 2024; Matos Fialho et al., 2021).

Moreover, this study also revealed a positive correlation between the loudness of the first perceived sound and anxiety levels, which was more pronounced in students with a lower propensity for anxiety, whereas the correlation between anxiety levels and overall acoustic satisfaction was more evident among students with higher anxiety tendencies. Furthermore, no significant correlations were found between the loudness of the second and third perceived sounds and anxiety levels. These results underscore the importance of the order of acoustic perception and suggest that the association between acoustic perception and anxiety differs among population groups.

Specifically, among different types of sound sources, although previous studies have shown that natural sounds are beneficial in reducing anxiety (Zhu et al., 2024), this study found that the loudness of natural sounds was positively correlated with anxiety levels. This phenomenon may be attributed to the interaction between natural sounds and contextual factors, such as pandemic-induced generalized anxiety, during the lockdown period, which diminished their positive attributes. Meanwhile, the loudness of electromechanical sounds exhibited the most pronounced negative correlation with anxiety levels. Therefore, attention should be given to controlling the loudness of both natural and electromechanical sounds.

However, the role of sound source types varies across different environments. For instance, in an office environment, project-related communication serves as a disturbing noise source, and speech noise can impact work efficiency (S. Kang et al., 2022). While university students on campus exhibited relatively low anxiety levels in response to social/communal sounds, voice and instrumental sounds were relatively low, suggesting a high tolerance towards these two sound sources. This may be attributed to the more relaxed social nature of university environments compared to office environments, and the pandemic-related information conveyed by these sounds may offer psychological comfort to individuals, as evidenced by the literature, which confirms the association between spending time obtaining information about COVID-19 and increased positive well-being (Fan & Smith, 2021). Therefore, in addition to the loudness of sound, the connotation of sounds is equally important.

Overall, our study highlights the relationship between acoustic perception and anxiety levels, emphasizing the importance of considering both the type and loudness of sound sources. These findings have significant implications for designing acoustic environments that can better support mental health, particularly in high-stress contexts such as the pandemic.

### 4.4. Applications

These findings serve as a critical step towards enhancing mental health support for university students and improving their overall well-being in the context of anxiety. By examining how university students perceive sound and its relationship with their anxiety levels, this study provides a foundation for sound adjustment to support mental well-being.

Despite the conclusion of the pandemic, the mental health of students remains in a state of crisis in the post-COVID-19 era (Lv et al., 2023). University studies represent a pivotal phase in life transition and social adaptation, where students encounter numerous pressures that constitute substantial challenges throughout their university experience (Labrague et al., 2017). In the stringent higher education environment, students often endure elevated levels of stress (Karyotaki et al., 2020). Consequently, university students have emerged as a high-risk group for stress issues (H. Wang et al., 2024). Evidence indicates that university students under stress are more likely to experience anxiety (Qin et al., 2023). Furthermore, learning is a factor that can cause anxiety, with most students experiencing persistent anxiety throughout their studies (Mofatteh, 2021).

Given the prevalence of stress and anxiety experienced by university students, this study examined the correlation between acoustic perception and anxiety to suggest potential interventions from an acoustic perspective. These strategies are anticipated to offer effective interventions for mitigating prevailing psychological issues among university students, thereby enhancing their mental health and overall well-being. For example, it is imperative to regulate the loudness of electromechanical and natural sounds, given the positive correlation between the loudness of these sound sources and anxiety levels.

Furthermore, understanding the connection between the acoustic environment and anxiety can inform the creation of an acoustic environment on campus that raises awareness of the importance of sounds in mental health, ultimately contributing to a more holistic approach to student well-being.

### 4.5. Limitations

Although this study provides valuable insights into the relationship between acoustic perception and anxiety among university students, it is not without limitations. One notable constraint is the geographical scope of this study, which, due to the influence of the pandemic, was confined to specific regions of China during the lockdown period. In addition, the study was conducted with limited factors, while variables such as socioeconomic status were not considered. This limitation underscores the need for future studies to ensure a more comprehensive understanding of acoustic perceptions and their relationship with anxiety across diverse settings and populations. Additionally, expanding the scope would allow the consideration of other potential factors that could affect the relationship between acoustic environments and mental health, such as architectural differences, socioeconomic status, cultural differences, and additional demographic variables. This broader approach contributes to a more robust and generalizable understanding of the interplay between acoustic environments and anxiety levels among university students.

## 5. Conclusions

The pandemic offered a unique large-scale quasi-experimental context to examine the correlation between acoustic perception and anxiety. By examining subjective perceptions of the acoustic environment, this study revealed a correlation between acoustic perception and anxiety levels. The main conclusions are summarised as follows:

(1) Respondents’ perceptions of the acoustic environment correspondingly varied during the lockdown; they reported a decreased perception of the less preferred motorised transport sounds, while the increased indoor noise of voice and instrumental caused annoyance. Concurrently, women exhibited higher sensitivity to variations in acoustic perception compared to men, especially concerning natural, voice and instrumental sounds. Moreover, psychoacoustic evaluations further suggested that the fluctuation strength was generally lower during the lockdown, with the overall acoustic environment being at a quieter level.

(2) Over half of the respondents indicated that they experienced anxiety symptoms during the lockdown. The majority of these were characterized by mild anxiety (32.4%), followed by moderate anxiety (13.6%), while the proportion of respondents with severe anxiety was the lowest (8%). Notably, those who were more sensitive to variations in the acoustic environment were more prone to experiencing anxiety symptoms and exhibited higher levels of anxiety. Moreover, while no statistically significant differences were observed in anxiety levels across sex, student type, and pandemic risk level categories, women (58.1%), undergraduates (55.7%), and respondents from high-risk areas (56.1%) were more susceptible to anxiety symptoms, especially in the group with mild anxiety.

(3) A notable correlation exists between acoustic perception and anxiety levels, although these correlations differ among the various student groups. Generally, a negative correlation was observed between the overall acoustic satisfaction and anxiety levels (*p* < 0.05), particularly for individuals who were more susceptible to anxiety, such as women, undergraduates, and respondents from high-risk areas. The importance of satisfaction with the acoustic environment is particularly pronounced among these groups. Conversely, no correlation was found between anxiety levels and overall acoustic satisfaction among students with a low proneness to anxiety. Furthermore, the loudness of the first perceived sound was positively correlated with anxiety levels (*p* < 0.01), this correlation was significantly stronger in groups less prone to anxiety, such as men. Moreover, the perception of different types of sound sources by respondents has varying relationships between anxiety levels. Specifically, the loudness of the electromechanical and natural sounds was positively correlated with anxiety, with the former being more significant (*p* < 0.01). Contrarily, the respondents exhibited a higher tolerance for the loudness of voices and instrumental, as well as social/communal sounds. However, no significant correlation was found between the loudness of the two sound sources and anxiety levels.

The results of this study provide insight into the correlation between acoustic perception and students’ mental health, particularly in relation to anxiety. The importance of this study lies in its contribution to a more nuanced understanding of how acoustic perception relates to students’ emotional states. It also suggests potential strategies for optimising the acoustic environment to mitigate psychological stress induced by emerging challenges or crises.

## Figures and Tables

**Table 1:** The equivalent continuous A-weighted sound pressure level (*L*_Aeq_) recorded at the selected locations during and after the lockdown. SD = standard deviation. All = the average of P1–P3 measurement locations, P1 = residential area, P2 = recreational area, P3 = traffic routes.

Locations	Time		*L*_Aeq_ (dB)	
			Mean	SD
All	During the lockdown	Morning	43.0	11.0
		Noon	42.9	11.2
		Night	52.2	8.8
	After the lockdown	Morning	56.0	6.7
		Noon	57.3	6.9
		Night	52.6	5.5
P1	During the lockdown	Morning	47.6	1.4
		Noon	53.7	1.8
		Night	63.8	1.4
	After the lockdown	Morning	58.6	1.4
		Noon	61.3	1.0
		Night	58.0	1.3
P2	During the lockdown	Morning	28.7	4.2
		Noon	28.0	2.9
		Night	44.5	3.9
	After the lockdown	Morning	47.8	1.5
		Noon	49.3	1.8
		Night	46.6	1.9
P3	During the lockdown	Morning	52.8	4.3
		Noon	47.0	2.8
		Night	48.2	2.5
	After the lockdown	Morning	61.7	4.7
		Noon	61.2	6.5
		Night	53.3	4.4

**Table 2:** The correlation between the loudness of the first perceived of different sound sources and anxiety levels. The numbers in parentheses are correlation coefficients, with significant correlations marked with * (*p* < 0.05) and ** (*p* < 0.01).

Psychological Indicators	Sound Sources
Anxiety levels	motorised transport (0.280)
	human movement (0.359)
	electromechanical (0.467 **)
	voice and instrumental (0.071)
	other human (0.090)
	social/communal (0.174)
	natural (0.339 *)
	other (0.306)

## Data Availability

The raw data supporting the conclusions of this article will be made available by the authors upon request.

## Supplementary Materials

The following supporting information can be downloaded at: https://www.mdpi.com/article/10.3390/bs15030262/s1. Questionnaire Survey Form.
