# Supplementary material for: Understanding the Nexus Between Anxiety and Acoustic Perception in University Students: A Quasi-Experimental Study During Pandemic-Induced Lockdown
Source: Behav Sci (Basel). 2025 Feb 24;15(3):262. doi: 10.3390/bs15030262 (PMC11939811; doi:10.3390/bs15030262)
Supplement: Supplementary file 1 [file behavsci-15-00262-s001.zip › behavsci-3343451-supplementary.pdf]

Contents of the questionnaire

Section	Question	type
1.Demographics	1: Name	(a)
	2: Sex	(b)
	3: Student types	(b)
	4: Address	(a)
2. The Generalised Anxiety Disorder 7-item scale (GAD-7)	1: Feeling nervous, anxious, or on edge	(c)
	2: Not being able to stop or control worrying	(c)
	3: Worrying too much about different things	(c)
	4: Trouble relaxing	(c)
	5: Being so restless that it is hard to sit still	(c)
	6: Becoming easily annoyed or irritable	(c)
	7: Feeling afraid as if something awful might happen	(c)
3. Acoustic perception	1: Please list the first perceived sound during the campus lockdown (limit one type)	(a)
	2: Please evaluate the loudness of the first perceived sound (1 = strongly small; 5 = very loud)	(c)
	3; Please evaluate the preference of the first perceived sound (1 = strongly dislike; 5 = very like)	(c)
	4: Please list the second perceived sound during the campus lockdown (limit one type)	(a)
	5: Please evaluate the loudness of the second perceived sound (1 = strongly small; 5 = very loud)	(c)
	6: Please evaluate the preference of the second perceived sound (1 = strongly dislike; 5 = very like)	(c)
	7: Please list the third perceived sound during the campus lockdown (limit one type)	(a)
	8: Please evaluate the loudness of the third perceived sound (1 = strongly small; 5 = very loud)	(c)
	9: Please evaluate the preference of the third perceived sound (1 = strongly dislike; 5 = very like)	(c)
	10: Please rate your overall acoustic satisfaction on campus (1 = very dissatisfied; 5 = very satisfied).	(c)
	11: Which sound sources have increased during the pandemic?	(a)
	12: Which sound sources have decreased during the pandemic?	(a)
	13: How has your emotional state changed after the lockdown? (1 = very bad; 5 = very good)	(c)
	14: During the lockdown, what sounds did you find most desirable?	(a)
	15: How quiet did you perceive the environment to be during the lockdown? (1 = very noisy; 5 = very quiet)	(c)

	16: During the lockdown, which sound did you find most pleasant?	(a)
	17: Please rate your liking for the most pleasant sound during the lockdown (1 = very dislike; 5 = very like)	(c)
	18: During the lockdown, which sound did you find most annoying? (1 = not annoying; 5 = very annoying)	(c)
	19: Please rate your annoyance with the most unpleasant sound during the lockdown (1 = not annoying; 5 = very annoying)	(c)
4. The impact of the pandemic lockdown	1: During the lockdown, which factor was most affected? (life, work, entertainment, others)	(b)
	2: How fearful do you find Covid-19? Please rate on a scale of 1 to 5 (1 = not fearful at all; 5 = very fearful)	(c)
	3: Where was your favorite place to visit before the pandemic?	(a)
	4: Where was your favorite place to visit after the pandemic?	(a)
	5: How has your acoustic environment changed before and after the pandemic-related lockdown?	(a)
	6: In what specific ways has the pandemic altered your life?	(a)
	7: Has the pandemic expanded your experiences in the virtual world (including all online activities)? Are you satisfied with this change?	(a)
